# Is tobacco a driver of footfall amongst small retailers? A geographical analysis of tobacco purchasing using electronic point-of-sale data

**DOI:** 10.1136/tobaccocontrol-2021-057089

**Published:** 2022-07-18

**Authors:** Helena Tunstall, Niamh K. Shortt, Amanda Y. Kong, Jamie Pearce

**Affiliations:** Centre for Research on Environment, Society and Health, Drummond Street, School of GeoSciences, The University of Edinburgh, Edinburgh, EH8 9XP, UK; Centre for Research on Environment, Society and Health, School of GeoSciences, The University of Edinburgh, Edinburgh, UK; Department of Family and Preventive Medicine, The University of Oklahoma Health Sciences Center, Oklahoma City, USA; TSET Health Promotion Research Center, Stephenson Cancer Center, The University of Oklahoma Health Sciences Center, Oklahoma City, USA; Centre for Research on Environment, Society and Health, School of GeoSciences, The University of Edinburgh, Edinburgh, UK

## Abstract

**Objective:**

Opponents of policies designed to reduce tobacco retail availability argue that tobacco products are a vital driver of ‘footfall’ in small retailers. This study considers the changing contribution of tobacco to footfall and revenue amongst convenience stores across Britain, compares tobacco to other ‘footfall driver’ products and assesses whether tobacco’s importance varies by neighbourhood deprivation and urban/rural status.

**Methods:**

We conducted an analysis of Electronic Point of Sales Systems (EPOS) data from 1,253 convenience stores in Britain in four weeks in 2016 and 2019. We calculated the number and value of purchased basket types (Tobacco Only, Non-Tobacco, Mixed) in each year and by neighbourhood characteristics.

**Results:**

The mean numbers of baskets per store containing tobacco fell by 47% during 2016–2019, a greater decline than any other footfall driver product. The sales value of tobacco products rose sharply over this time period due to increasing unit price. However, the proportion of store turnover accounted for by tobacco transactions declined. There were marked falls in the turnover from non-tobacco products in Mixed tobacco baskets. The proportion of baskets containing tobacco and the value of turnover from these baskets was greater in more deprived and urban areas but these places also experienced larger reductions over time, narrowing differences between areas.

**Conclusion:**

Tobacco’s importance as a driver of footfall and related turnover among convenience retailers has reduced significantly in Britain in recent years, particularly in deprived and urban areas, undermining industry claims that tobacco is essential to the viability of these businesses.

## INTRODUCTION

Reducing the availability of tobacco products is increasingly seen by tobacco control advocates as a policy priority to lower the prevalence of smoking and address health inequalities [1–4]. For example, in 2021 the New Zealand Government published a Smokefree Action Plan that included proposals to make tobacco products less available by reducing the number of retailers permitted to sell tobacco [5]. The Scottish Government has also identified reductions in the availability of tobacco as a potential area for policy development in the next phase of its tobacco control strategy [6]. Tobacco manufacturers have sought to undermine these efforts by arguing that tobacco plays a central role in the business of small retailers and therefore is essential to their viability [7]. These arguments, presented in industry publications and the retailer trade press [8, 9], stress the importance of tobacco in both attracting footfall and increasing wider expenditure in small retailers [7].

This study focuses upon sales of tobacco in convenience stores in Britain. There are currently approximately 50,000 convenience stores across the country [10] and these accounted for 55–60% of the total volume of cigarettes purchased during 2007–2015 [11]. These small retailers sell a variety of grocery items in addition to tobacco products, including snack foods, confectionery, lottery tickets, alcohol, soft drinks, household goods, newspapers and magazines, to a mostly local catchment population [12].

There is some analysis that has attempted to quantify the importance of tobacco to convenience store businesses which indicates that tobacco has played a significant role as a ‘traffic builder’ among small retailers [8]. Tobacco has been one of the products most commonly cited as motivating a visit to a convenience store in the UK [13]. Recent research has, however, begun to challenge some aspects of the tobacco industry narrative regarding the role of tobacco footfall in small retail businesses [7, 14]. Consumer intercept surveys of shoppers exiting stores in case study areas in USA, New Zealand and Australia have found that just 8–14% of transactions contained tobacco [14–17]. Additionally, tobacco shoppers did not necessarily make significant ‘secondary purchases’ as 60–64% of these shoppers bought tobacco alone [15–17]. A 2015 analysis of point-of-sale data in the UK indicated that tobacco was purchased by a minority of shoppers, was often purchased alone, and the value of any non-tobacco co-purchases was similar to that purchased by shoppers who did not buy tobacco [7].

Whilst there is some nascent work on tobacco-generated footfall, to date there has been little analysis of the spatial variation of tobacco sales and how the importance of such sales to small retailers varies by neighbourhood factors, such as deprivation and urban and rural status. Better knowledge of how the financial role of tobacco varies between stores in different types of neighbourhoods is important to the development of policies intended to reduce spatial concentrations of tobacco retailing. Understanding these factors in socioeconomically deprived areas is particularly pertinent because these areas have greater numbers of tobacco outlets [18–23].

Further, few studies have undertaken a robust assessment of tobacco and co-purchases based on electronic point-of-sale (EPOS) transaction data. A recent geographical analysis of UK EPOS data has shown the value of these data, demonstrating that whilst tobacco sales were greater in areas of higher deprivation, gross margins on tobacco were lower here than in other areas [24], likely reflecting greater sales of cheaper tobacco brands [25].

This study builds on this earlier work by assessing how the importance of tobacco in driving footfall, secondary purchasing and sales turnover of convenience retailers in Britain is changing over time, using data from store tills in 2016 and 2019. It also considers where tobacco footfall is most important, comparing stores in areas with different levels of deprivation and urban/rural status, and analyses how these spatial patterns have changed over the study period.

More specifically, the research addresses the following questions:

How have patterns of sales of tobacco in convenience stores in Britain changed between 2016 and 2019, in terms of:

- the proportion of shopping baskets containing tobacco;
- the sales value of products in tobacco baskets (tobacco and non-tobacco);
- the proportion of total store turnover accounted for by products in tobacco baskets (tobacco and non-tobacco);
- differences in these retail outcomes between stores in more and less deprived areas and rural and urban areas?

## METHODS

### Tobacco retail data

EPOS data were provided by The Retail Data Partnership (TRDP) who supply their till system ‘ShopMate’ to a selection of convenience stores across Britain. TRDP clients include fully independent stores and stores which are members of a form of franchise known as ‘symbol group’ in the UK (e.g. Premier, Londis and Costcutter). These retailers usually own and operate their own stores [26] but are affiliated with a symbol group company, which provides the store with products, services, and a branded shop fascia [27]. The data describes purchases that were scanned by barcode on the stores’ EPOS. This sales data includes the barcode, product name, number, type and sales value of products purchased by each customer and a store identifier. For this study, TRDP provided a data extract from the weeks of 7–13 March, 7–13 June, 7–13 September and 7–13 December in 2016 and 2019. These weeks were selected to represent purchasing patterns across the seasons, outside major school holidays.

### Case selection

TRDP provided purchasing data on 1,360 stores located in Britain which recorded one or more tobacco sales in all study weeks in 2016 and 2019. Stores which reported limited hours of transactions, suggesting they may not have been fully open or recorded all of their sales on their TRDP EPOS, were excluded from the analysis. A total of 107 stores were excluded as they reported no sales of any product types on one or more days Monday through Friday (N=95), or recorded sales over a span of less than two hours on a day when sales were recorded (N=16), in one or more study weeks, resulting in an analytic sample of 1,253 eligible convenience stores in the current study. The sample contained 390 independent stores and 863 stores that were members of symbol group franchises and comprised 939, 129 and 195 stores in England, Scotland and Wales respectively (Supplementary Table A).

### Geographical variables

TRDP linked the postcode location of the convenience stores to neighbourhood measures of income deprivation and urban/rural status. Neighbourhoods were defined by Lower Super Output Areas (LSOAs) in England and Wales and Data Zones in Scotland, areas with populations of approximately 1,700 and 800 residents respectively in 2016 [28, 29].

Neighbourhood deprivation was measured by the Income Domain of the English Indices of Deprivation (IMD) 2019 [30], the Welsh Index of Deprivation (WIMD) 2019 [31] and the Scottish Index of Multiple Deprivation (SIMD) 2020 [32]. The Income Domain is based upon the proportion of the neighbourhoods’ population in receipt of means tested state benefits. Stores were grouped into income deprivation quintiles (1=least deprived; 5=most deprived), based on their neighbourhood income deprivation rank within each country. The most deprived quintile contained the fifth most deprived LSOAs in England, the fifth most deprived LSOAs in Wales and the fifth most deprived Data Zones in Scotland.

Neighbourhood urban/rural status was indicated by a hierarchy of four categories: ‘Large Urban’, ‘Other Urban’, ‘Town Rural’ and ‘Village Rural’. We created these categories by combining the four hierarchical categories of the England and Wales Rural Urban Classification 2011 for Lower Super Output Areas [33], ‘Major and Minor Conurbation’, ‘City and Town’, ‘Town and Fringe’ and ‘Village’ respectively, and the four hierarchical categories of the Scottish Government Urban Rural Classification 2016 [34], ‘Large Urban Areas’, ‘Other Urban Areas’, ‘Small Towns’ and ‘Rural Areas’ respectively. The categories ‘Large Urban’ and ‘Other Urban’ identify ‘urban’ neighbourhoods located in settlements with populations greater than 10,000 and ‘Town Rural’ and ‘Village Rural’ are in ‘rural’ settlements with populations below this threshold.

### Defining ‘footfall driver’ products

The analysis compares tobacco leaf products (hereafter termed ‘tobacco’), including cigarettes, roll your own (RYO), cigar, pipe and heated tobacco products (‘heat-not-burn’ tobacco), to other products that may attract footfall to convenience stores. Smoking accessories (including tobacco papers, lighters and matches) and electronic-cigarettes (including vape devices and liquids) were also included, to provide a full picture of tobacco-related products purchased. The ‘footfall driver’ products selected for analysis included the six most frequently cited by shoppers as reasons for visiting a UK convenience store which were, in addition to tobacco, milk, soft drinks, newspapers and magazines, bread and sandwiches [13]. The analysis also considered snacks and confectionery, commonly cited as motivating store visits among ages 16–34 years, and alcohol, fresh fruit and vegetables and fresh meat and fish, cited at ages 35 years and older [13]. Finally, lottery tickets were assessed because of their potential role in attracting new customers and encouraging regular weekly store visits [35].

The tobacco products, with the exception of heated tobacco products, were defined using EPOS derived product categories. The remaining product categories were defined using either EPOS derived categories alone or in combination with text searches of product names.

### Purchasing measures

A ‘basket’ refers to the group of items purchased in one transaction by a single customer. The retail data does not contain direct information on the motivation for store visits, so in this analysis the potential role of tobacco products in driving footfall and secondary purchasing was indicated by counts of baskets containing tobacco and assessment of the value of non-tobacco products purchased by tobacco shoppers, The analyses focusses on three basket types: ‘Tobacco Only’ (baskets only containing tobacco products), ‘Mixed’ (baskets containing tobacco and non-tobacco products), and ‘Non-Tobacco’ (baskets only containing non-tobacco products). A ‘Tobacco Basket’ is a transaction containing one or more tobacco products, either a Tobacco Only or Mixed basket.

The potential financial importance of tobacco footfall was considered by assessing tobacco ‘basket sales values’ and weekly ‘store turnover’. ‘Basket sales value’ describes the sales value of tobacco and non-tobacco items within individual baskets. The sales value of tobacco and non-tobacco products described in this analysis include all required taxes and duties. Weekly ‘store turnover’ describes the total sales value of tobacco and non-tobacco items sold in a week within stores.

The analysis was stratified by basket type (Tobacco Only, Mixed, Non-Tobacco), and product type (Tobacco, Non-Tobacco), time period (2016, 2019, Change 2016–2019) and area deprivation and urban/rural status. The main outcomes assessed were the percentages of baskets containing tobacco, the mean basket sales values of tobacco baskets and the percentage of total weekly turnover from tobacco baskets. We fit linear regression analysis models to assess the relationship between stores’ deprivation level and urban/rural status and the stores’ percentages of baskets containing tobacco in 2016 and 2019, as well as the stores’ percentage of change in baskets containing tobacco between 2016 and 2019.

## RESULTS

### Volume of total baskets, basket size and total turnover

Between 2016 and 2019, the mean number of baskets purchased per store in the study weeks declined by 16.2%, from 2,262 to 1,896 (Table 1). While the size of baskets increased by 6.2% from a mean of 2.61 to 2.77 items, the total value of weekly sales turnover fell by 1.1% from £10,785 to £10,665. These results were broadly consistent across deprivation and urban/rural categories, but with relatively larger declines in numbers of basket purchased and turnover in Large Urban areas and bigger increases in basket size in the most deprived areas.

### Volume of baskets containing footfall driver products

The proportion of total baskets containing tobacco products fell from 24.3% in 2016 to 15.3% in 2019, representing a mean of 549 and 291 baskets per week respectively, and a relative decline in basket numbers of 47.0% (Table 2). The fall in numbers of baskets containing tobacco was greater than for any other footfall driver products, but other footfall products also saw significant declines, including newspapers and magazines (−24.6%), soft drinks (−10.1%), confectionery (−9.4%) and bread (−9.2%). The small number of product types that experienced growth included e-cigarettes (171.7%), smoking accessories (2.0%), alcopops (32.5%) and spirits (8.6%)

### Volume of tobacco baskets

In 2016, 10.6% of all baskets purchased were Tobacco Only, but this declined to 6.2% in 2019 (Table 3). The proportion of baskets containing tobacco and non-tobacco products (Mixed) also declined from 13.7% to 9.1%. In contrast, the proportion of baskets that contained only non-tobacco products (Non-Tobacco) increased from 75.7% to 84.7%, representing an 8.9% increase. The proportion of tobacco shoppers that purchased only tobacco was 43.6% in 2016 but declined to 40.5% in 2019.

Comparison by neighbourhood type (Table 3) indicates that the proportion of baskets containing tobacco increased as a gradient between the most and least deprived and urban areas in 2016 and 2019, with stores in the most deprived and urban areas recording the largest proportion of Tobacco Baskets, both Tobacco Only and Mixed. Between 2016 and 2019, all areas experienced substantial falls in the proportion of Tobacco Baskets; however, there were greater absolute and relative falls in more deprived and urban areas.

In 2016 the proportion of baskets that contained tobacco in the most deprived quintile was 1.34 times greater than in the least deprived quintile, but by 2019 this ratio had reduced to 1.22. The proportion of tobacco baskets in Large Urban areas was 1.55 times greater than in Village Rural areas in 2016, reducing to 1.39 times greater by 2019.

Linear regression model results (Supplementary Table B) indicated that both deprivation and urban/rural status were independently related to the percentage of stores’ baskets that contained tobacco in 2016. Analysis of change between 2016 and 2019 indicated that there were significantly larger falls in percentage of stores’ baskets that contained tobacco, both in more deprived and urban areas. In 2019 the percentage of stores’ baskets that contained tobacco was still significantly elevated in urban areas relative to Village Rural but high deprivation was no longer independently associated with an elevated percentage of tobacco baskets relative to the least deprived quintile.

### Sales value of tobacco baskets

The mean basket value of tobacco in Tobacco Only and Mixed baskets rose markedly between 2016 and 2019, increasing by 63.1% and 64.9% respectively (values not adjusted for inflation) (Table 4), while the mean basket value of non-tobacco products in Mixed tobacco baskets and Non-Tobacco baskets increased by a fifth. Mean values of tobacco in Tobacco only and Mixed tobacco baskets were lowest in the most deprived and urban areas in 2016 but increased faster in these areas, narrowing the difference in mean tobacco basket spend between area types by 2019.

### Weekly turnover from tobacco baskets

Between 2016 and 2019, the proportion of total weekly store turnover that came from tobacco baskets fell 7.8%, from 47.0% to 39.2% (Table 5). The declining role of tobacco sales contributed to this fall, with the proportion of turnover accounted for by Tobacco Only baskets falling from 15.5% to 12.5% and tobacco in Mixed baskets from 20.2% to 18.9% during 2016–2019. However, the importance of non-tobacco products in Mixed tobacco baskets also declined significantly, reducing from 11.3% of total turnover in 2016 to only 7.8% in 2019, a relative decline of 31%.

The proportion of total store weekly turnover that came from Tobacco Only and Mixed baskets was greater among stores in more deprived places compared to less deprived areas in 2016 and 2019. Over this time period, however, the proportion of total turnover that came from these basket types declined further in more deprived areas, narrowing the difference between the most and least deprived areas. Conversely, there was an absolute increase in the proportion of total turnover from baskets containing only non-tobacco products of 9.1% in the most deprived quintile compared to 5.5% in the least deprived quintile. Similarly, in Large Urban and Other Urban areas the proportion of store turnover from Tobacco Only and Mixed baskets was greater compared to more rural areas in 2016 and 2019. However, over this time period the proportion of turnover from tobacco baskets fell further in more urban areas, narrowing differences between urban and rural stores.

## DISCUSSION

### Declining tobacco transactions and associated secondary purchasing in convenience stores

Between 2016 and 2019, the number of transactions which involved tobacco fell by almost half in convenience stores in Britain, declining significantly more than any other footfall driver products that were analysed. While the purchase cost of tobacco products was substantially more expensive in 2019 than in 2016, the contribution of tobacco baskets to total turnover declined markedly. Counter to arguments that tobacco products maintain a vital role encouraging secondary purchases, we found that the proportion of total store turnover accounted for by non-tobacco products purchased by tobacco shoppers decreased by almost a third. Conversely, the proportion of store turnover accounted for by shoppers who did not purchase tobacco rose significantly.

During 2016–2019, the proportion of adults in the UK who were current smokers fell from 15.8% to 14.1% [36], and smokers’ daily cigarette consumption in England declined from 11.2 to 10.6 cigarettes, from 2016 to 2018 [37]. Our study covered a time period that included significant changes to the regulation of tobacco sales in UK, which is likely to have contributed to the observed declines in tobacco footfall. In May 2017 the EU Revised Tobacco Products Directive on standardised packaging and minimum pack size laws came into full effect across the UK. This banned sales of smaller packets of cigarettes of fewer than 20 sticks and RYO packs of less than 30g. This policy required consumers to purchase tobacco in large pack sizes which may have resulted in fewer trips to stores to purchase tobacco products. The ban on small packets of cigarettes is likely to have had greater impact on small stores than larger retailers because more of their sales had been small packs of 10 and 14–19 sticks [11]. A decline in the number of trips to convenience stores made by tobacco shoppers may in turn have reduced their spending on non-tobacco products.

### Tobacco footfall in deprived and urban neighbourhoods

This study found that in the most deprived neighbourhoods more baskets contained tobacco products, which is consistent with earlier work in the UK [24]. Importantly however, the current paper included a temporal analysis and the results indicate that this pattern is weakening, as the number of tobacco transactions declined faster in more deprived places.

High levels of tobacco transactions in deprived areas are likely to reflect, in part, the smoking prevalence amongst local populations [24, 38–42]. In the last decade smoking prevalence among adults has declined across all levels of deprivation in Britain [39–42]. Some data for the time period of this study does indicate there were relatively greater falls in smoking rates in more deprived places [39] but other evidence contradicts this [40, 42], suggesting trends in smoking prevalence may not account for the large falls found in tobacco transactions in deprived areas.

In this analysis, deprived areas had greater increases in the mean sales value of tobacco products within tobacco baskets between 2016 and 2019. This suggests that following the implementation of the ban on small packs, more shoppers in deprived areas switched to larger packs, which were more expensive and purchased less frequently.

The study finds that the value of turnover from tobacco shoppers’ baskets fell more sharply in deprived areas, suggesting that the importance of tobacco to the business models of retailers in these areas is waning. The possibility that tobacco control policies which limit tobacco sales may pose more risks in deprived areas both to small retailers, who may be more financially dependent on tobacco sales, and their customers, who may be more dependent on the availability of these small local stores for their groceries [43, 44], may also be receding.

The analysis also found that numbers of baskets containing tobacco fell faster in urban areas in comparison to more rural places. In rural areas, with limited retail accessibility, tobacco shoppers may be more likely to ‘stock up’ in store by buying large packs of tobacco [45] and so their purchasing may have been less affected by restrictions on small tobacco packs.

### The future of tobacco footfall in small retailers

Studies of small retailers’ attitudes towards selling tobacco find many support arguments regarding tobacco’s importance in attracting customers and driving secondary purchases [9, 46–50]. Surveys in England have suggested small retailers are often concerned by the small profit margins associated with tobacco and the costs of tobacco stock, but most do not want to reduce their tobacco sales [7, 9, 49]. When small retailers have voluntarily disinvested from tobacco, common reasons cited were low sales and profits [50–53]. The large declines in tobacco transactions that this analysis describes may convince some small retailers that the financial impact of ceasing to sell tobacco is unlikely to be as significant as suggested by the tobacco industry.

Small retailers that have stopped selling tobacco frequently indicate that this decision was stimulated by changes to their business conditions, often following new legislation [52]. Legislative action, such as requiring an annual license fee to sell tobacco products [54], may prompt some retailers to reconsider the financial value of tobacco [53]. Tobacco control policies could also be designed to support small retailers who wish to voluntarily end tobacco sales, through financial incentives [53].

Early evidence suggests that the pattern of declining tobacco transactions in convenience stores in Britain found in this study during 2016–2019 came to an abrupt halt in 2020 with the arrival of the Covid-19 global pandemic [55, 56]. The longer-term implications of the pandemic for tobacco purchasing in small retailers, including the impact on footfall, is hard to predict and deserves future attention.

### Methodological strengths and limitations

This study benefits from the use of retail data containing comprehensive and accurate records of transactions scanned in convenience stores through TRDP EPOS. However, these data omit the minority of store sales that were entered on the TRDP EPOS using ‘hotkeys’ or were recorded on other tills. Hotkeys are used to increase speed of sales of popular items and to enter sales of items that do not have a barcode or regular price. The omission of hotkey sales is likely to have had relatively little impact on recorded tobacco sales, as tobacco product sales are usually entered on EPOS with barcodes. This omission will have had a greater effect on data for other footfall driver products analysed. Some tobacco baskets defined as ‘Tobacco Only’ in this analysis may have included non-tobacco products that were missing from the data because they were entered on the EPOS with hotkeys. The absence of hotkey sales also means that the turnover data assessed is incomplete.

Turnover is an important measure of store finances, but does not directly indicate business profits, which may be more pertinent to stores’ financial viability and retailers’ decisions regarding sales of tobacco products. The turnover figures presented have not been adjusted for inflation, but the changing proportion of turnover accounted for by tobacco baskets still provides an indication of how the financial value of tobacco has altered over time.

The stores in the study were an opportunity sample of TRDP clients and so were not selected to be representative of convenience stores selling tobacco across Britain. The significance of tobacco footfall is also likely to differ between retailer types [14].

These retail data do not include information about customers and so could not capture shoppers’ characteristics, motivations for visiting stores or frequency of purchasing. In the analysis, baskets containing tobacco were presented as an indicator of tobacco’s role as a footfall driver and non-tobacco products purchased alongside tobacco were presumed to be ‘secondary purchases’. However, tobacco in mixed baskets may have been an incidental purchase made by shoppers whose store visit was motivated by other types of products. This analysis therefore may overestimate the importance of tobacco as a driver of footfall and related secondary purchasing.

The use of product name text searches to define product categories will have resulted in imprecision in the classifications of some products. The analysis combines three income deprivation ranking indicators that were defined separately for England, Wales and Scotland, countries which have different levels of deprivation, and combined two urban/rural indicators for England and Wales and Scotland that were also defined in separate and distinct ways.

## CONCLUSION

The tobacco industry has long presented the sale of tobacco products as essential to the survival of small retailers because of its role in attracting footfall and driving wider purchasing [7]. The rapid decline in the numbers of transactions involving tobacco and in associated secondary purchases in convenience stores in Britain has weakened these arguments and suggests an opportunity for tobacco control policies to support retailers in divesting from tobacco sales.

## Supplementary Material

Online Supplement

## Figures and Tables

**Table 1.** Numbers of stores, baskets and items sold by area deprivation and urban/rural status, Britain (2016, 2019)

Area type	Stores (N)	Mean baskets per store per week			Mean items per basket			Mean turnover per store per week		
		2016 (N)	2019 (N)	Change 2016–2019 (%)	2016 (N)	2019 (N)	Change 2016–2019 (%)	2016 (£)	2019 (£)	Change 2016–2019 (%)
**Total**	1,253	2,262	1,896	−16.2	2.61	2.77	6.2	10,785	10,665	−1.1

**Income Deprivation**										
1 Least deprived	120	1,740	1,456	−16.3	2.48	2.59	4.4	8,114	7,914	−2.5
2	186	1,874	1,623	−13.4	2.58	2.72	5.1	9,214	9,329	1.3
3	246	2,077	1,754	−15.5	2.65	2.78	5.1	10,172	10,099	−0.7
4	304	2,330	1,961	−15.8	2.62	2.78	6.3	11,285	11,237	−0.4
5 Most deprived	397	2,663	2,194	−17.6	2.62	2.81	7.3	12,326	12,035	−2.4

**Urban Rural Status**										
Large Urban	272	2,456	2,000	−18.6	2.54	2.70	6.4	11,508	11,070	−3.8
Other Urban	676	2,330	1,950	−16.3	2.60	2.77	6.4	11,062	10,938	−1.1
Town Rural	170	2,187	1,878	−14.2	2.68	2.83	5.6	10,668	10,744	0.7
Village Rural	135	1,621	1,435	−11.5	2.78	2.90	4.1	8,092	8,381	3.6

**Table 2.** Percentages of baskets containing footfall products, Britain (2016, 2019)

Product type	Mean baskets per store per week			Percentage of total baskets		
	2016 (N)	2019 (N)	Change 2016–2019 (%)	2016 (%)	2019 (%)	Change 2016–2019 (%)
Total	2,261.6	1,895.9	−16.2	100.0	100.0	0.0

Non-Tobacco	1,713.1	1,605.3	−6.3	75.7	84.7	8.9

Tobacco	548.5	290.6	−47.0	24.3	15.3	−8.9
Cigarettes	436.3	234.6	−46.2	19.3	12.4	−6.9
RYO	115.0	53.6	−53.4	5.1	2.8	−2.3
Cigar	5.2	4.1	−20.5	0.2	0.2	0.0
Pipe	1.0	0.4	−61.7	0.0	0.0	0.0
Heated tobacco products	0.0	0.2	-	0.0	0.0	0.0
News & magazines	273.1	206.0	−24.6	12.1	10.9	−1.2
News	238.1	179.6	−24.6	10.5	9.5	−1.1
Magazines	46.6	35.1	−24.6	2.1	1.9	−0.2
Soft drinks	608.7	547.0	−10.1	26.9	28.8	1.9
Confectionery	498.7	451.9	−9.4	22.1	23.8	1.8
Bread	109.9	99.8	−9.2	4.9	5.3	0.4
Fresh fruit & veg	22.7	20.7	−8.8	1.0	1.1	0.1
Milk (dairy unflavoured)	172.4	159.6	−7.5	7.6	8.4	0.8
Fresh meat & fish	60.9	57.2	−6.2	2.7	3.0	0.3
Snacks	250.0	240.7	−3.7	11.1	12.7	1.6
Alcohol	223.2	216.4	−3.1	9.9	11.4	1.5
Beer	114.7	108.5	−5.4	5.1	5.7	0.6
Cider	54.2	55.5	2.4	2.4	2.9	0.5
Alcopop	10.0	13.3	32.5	0.4	0.7	0.3
Spirit	46.3	50.3	8.6	2.0	2.7	0.6
Wine	62.7	58.4	−7.0	2.8	3.1	0.3
Lottery	220.3	221.2	0.4	9.7	11.7	1.9
Sandwiches	27.0	27.4	1.6	1.2	1.4	0.3
Smoking accessories	82.3	84.0	2.0	3.6	4.4	0.8
E-cigarettes	2.8	7.6	171.7	0.1	0.4	0.3

**Table 3.** Percentages of basket types by area deprivation and urban/rural status, Britain (2016, 2019)

Area type	Stores (N)	Total Tobacco baskets						Mixed Tobacco baskets			Tobacco Only baskets						Total baskets all four weeks	
		Mean baskets per store per week			Percentage of total baskets			Percentage of total baskets			Percentage of total baskets			Percentage of tobacco baskets				
		2016 (N)	2019 (N)	Change 2016–2019 (%)	2016 (%)	2019 (%)	Change 2016–2019 (%)	2016 (%)	2019 (%)	Change 2016–2019 (%)	2016 (%)	2019 (%)	Change 2016–2019 (%)	2016 (%)	2019 (%)	Change 2016–2019 (%)	2016 (N)	2019 (N)
**Total (N)**	1,253	548.5	290.6	−47.0	24.3	15.3	−8.9	13.7	9.1	−4.6	10.6	6.2	−4.4	43.6	40.5	−3.1	11,335,092	9,502,166

**Income Deprivation**																		
1 Least deprived	120	337.6	188.9	−44.0	19.4	13.0	−6.4	10.9	7.5	−3.4	8.5	5.4	−3.1	43.7	41.9	−1.9	834,965	698,973
2	186	405.2	231.1	−43.0	21.6	14.2	−7.4	12.0	8.4	−3.6	9.7	5.8	−3.8	44.7	41.0	−3.6	1,394,400	1,207,565
3	246	475.6	261.7	−45.0	22.9	14.9	−8.0	13.1	9.0	−4.1	9.8	6.0	−3.9	42.9	39.9	−3.0	2,043,712	1,726,194
4	304	590.0	317.6	−46.2	25.3	16.2	−9.1	14.3	9.6	−4.6	11.1	6.6	−4.5	43.7	40.5	−3.1	2,832,944	2,384,912
5 Most deprived	397	692.9	346.4	−50.0	26.0	15.8	−10.2	14.7	9.4	−5.3	11.3	6.3	−5.0	43.5	40.2	−3.3	4,229,071	3,484,522

**Urban Rural Status**																		
Large Urban	272	655.3	333.2	−49.2	26.7	16.7	−10.0	14.5	9.5	−4.9	12.2	7.1	−5.1	45.8	42.9	−2.9	2,672,068	2,176,383
Other Urban	676	579.7	302.9	−47.7	24.9	15.5	−9.3	13.9	9.2	−4.7	10.9	6.3	−4.6	44.0	40.7	−3.2	6,300,380	5,274,151
Town Rural	170	467.4	267.1	−42.8	21.4	14.2	−7.1	12.7	8.9	−3.9	8.6	5.4	−3.3	40.4	37.8	−2.6	1,487,405	1,276,715
Village Rural	135	279.5	172.5	−38.3	17.2	12.0	−5.2	11.0	8.0	−3.1	6.2	4.1	−2.1	36.0	33.8	−2.2	875,239	774,917

Note: Tobacco Baskets included tobacco products (Mixed or Tobacco Only); Mixed baskets included baskets that contained both tobacco products and other non-tobacco products; Tobacco Only baskets included baskets that contained only tobacco products; Non-Tobacco baskets included baskets that contained only non-tobacco products.

**Table 4.** Mean basket sales value by area deprivation and urban/rural status, Britain (2016, 2019)

Area type	Total baskets			Total Tobacco baskets			Mixed Tobacco baskets						Tobacco Only baskets			Non-Tobacco baskets		
	Tobacco & Non-tobacco			Tobacco & Non-tobacco			Tobacco			Non-tobacco			Tobacco			Non-tobacco		
	2016 (£)	2019 (£)	Change 2016–2019 (%)	2016 (£)	2019 (£)	Change 2016–2019 (%)	2016 (£)	2019 (£)	Change 2016–2019 (%)	2016 (£)	2019 (£)	Change 2016–2019 (%)	2016 (£)	2019 (£)	Change 2016–2019 (%)	2016 (£)	2019 (£)	Change 2016–2019 (%)
**Total**	4.77	5.63	18.0	9.24	14.40	55.8	7.05	11.63	64.9	3.95	4.83	22.4	6.98	11.38	63.1	3.34	4.04	21.0

**Income Deprivation**																		
1 Least deprived	4.66	5.43	16.5	9.79	14.76	50.8	7.59	12.03	58.5	4.03	4.87	21.0	7.44	11.78	58.5	3.43	4.04	17.9
2	4.92	5.75	16.9	9.85	14.94	51.6	7.56	12.06	59.4	4.10	4.93	20.3	7.60	11.98	57.6	3.56	4.22	18.8
3	4.90	5.76	17.5	9.65	14.75	53.0	7.37	11.94	61.9	4.12	4.94	19.9	7.19	11.56	60.8	3.49	4.18	19.8
4	4.84	5.73	18.3	9.30	14.41	55.0	7.10	11.66	64.3	3.93	4.79	22.1	7.08	11.41	61.3	3.33	4.05	21.6
5 Most deprived	4.63	5.48	18.5	8.79	14.00	59.2	6.66	11.25	68.9	3.83	4.76	24.2	6.58	11.00	67.2	3.16	3.89	22.9

**Urban Rural Status**																		
Large Urban	4.69	5.53	18.1	8.76	13.72	56.6	6.71	11.09	65.2	3.81	4.69	23.0	6.68	10.98	64.3	3.20	3.90	21.7
Other Urban	4.75	5.61	18.1	9.14	14.39	57.5	6.97	11.66	67.2	3.89	4.78	22.8	6.94	11.42	64.5	3.29	3.99	21.2
Town Rural	4.88	5.72	17.3	10.01	15.04	50.2	7.53	12.01	59.6	4.21	5.05	19.9	7.46	11.70	57.0	3.48	4.18	19.9
Village Rural	4.99	5.84	17.0	10.99	15.87	44.4	8.19	12.48	52.5	4.44	5.26	18.6	8.09	12.20	50.8	3.74	4.47	19.5

**Table 5.** Weekly turnover of basket types by area deprivation and urban/rural status, Britain (2016, 2019)

Area type	Percentage of store turnover															Total turnover all four weeks	
	Total Tobacco baskets			Mixed Tobacco baskets						Tobacco Only baskets			Non-Tobacco baskets				
	Tobacco & Non-tobacco			Tobacco			Non-tobacco			Tobacco			Non-tobacco				
	2016 (%)	2019 (%)	Change 2016–2019 (%)	2016 (%)	2019 (%)	Change 2016–2019 (%)	2016 (%)	2019 (%)	Change 2016–2019 (%)	2016 (%)	2019 (%)	Change 2016–2019 (%)	2016 (%)	2019 (%)	Change 2016–2019 (%)	2016 (£)	2019 (£)
**Total**	47.0	39.2	−7.8	20.2	18.9	−1.4	11.3	7.8	−3.5	15.5	12.5	−2.9	53.0	60.8	7.8	54,056,446	53,452,468

**Income Deprivation**																	
1 Least deprived	40.7	35.2	−5.5	17.8	16.7	−1.1	9.4	6.8	−2.7	13.5	11.8	−1.8	59.3	64.8	5.5	3,894,941	3,798,630
2	43.3	37.0	−6.3	18.4	17.6	−0.8	10.0	7.2	−2.8	14.9	12.2	−2.8	56.7	63.0	6.3	6,855,171	6,940,909
3	45.1	38.2	−6.9	19.7	18.6	−1.1	11.0	7.7	−3.3	14.4	12.0	−2.5	54.9	61.8	6.9	10,009,423	9,937,589
4	48.6	40.7	−7.9	20.9	19.6	−1.3	11.6	8.1	−3.5	16.2	13.1	−3.1	51.4	59.3	7.9	13,722,666	13,664,372
5 Most deprived	49.4	40.3	−9.1	21.2	19.4	−1.8	12.2	8.2	−4.0	16.1	12.7	−3.4	50.6	59.7	9.1	19,574,245	19,110,968

**Urban Rural Status**																	
Large Urban	49.9	41.3	−8.6	20.7	19.1	−1.6	11.8	8.1	−3.7	17.4	14.2	−3.3	50.1	58.7	8.6	12,520,767	12,044,693
Other Urban	47.9	39.9	−8.0	20.5	19.1	−1.3	11.4	7.8	−3.6	16.0	12.9	−3.1	52.1	60.1	8.0	29,911,824	29,575,894
Town Rural	43.8	37.4	−6.5	19.7	18.6	−1.1	11.0	7.8	−3.2	13.2	11.0	−2.2	56.2	62.6	6.5	7,254,397	7,305,886
Village Rural	38.0	32.7	−5.3	18.1	17.0	−1.1	9.8	7.2	−2.6	10.1	8.5	−1.6	62.0	67.3	5.3	4,369,458	4,525,996

Note: Proportion of store turnover describes the percentage of the stores’ total weekly recorded sales accounted for by items in baskets of each type.

## KEY MESSAGES

### What is already known on this topic

- Tobacco control strategies have increasingly focussed on reducing the availability of tobacco products, but tobacco manufacturers have responded by arguing that tobacco ‘footfall’ is essential to the viability of many small retailers.

### What this study adds

- Analysis of electronic point of sale data in small retailers in Britain in 2016 and 2019 indicated that the number of transactions containing tobacco fell by nearly half, declining by a greater proportion than any other ‘footfall driver’ product.
- The price of tobacco products rose significantly over this time period but the proportion of total store turnover accounted for by tobacco shoppers’ purchases of tobacco and non-tobacco products fell, declining most in more deprived and urban areas where tobacco transactions had been greatest.

### How this study might affect policy

- As the financial importance of tobacco to small retailers across Britain has decreased, the tobacco manufacturers’ arguments that tobacco footfall is vital to these businesses have weakened and the potential for policies that encourage small retailers to end tobacco sales to contribute to tobacco control is growing.
