# Supplementary material for: Is tobacco a driver of footfall amongst small retailers? A geographical analysis of tobacco purchasing using electronic point-of-sale data
Source: Tob Control. Author manuscript; Available in PMC 2024 Jan 18. (PMC10344415; doi:10.1136/tobaccocontrol-2021-057089)
Supplement: Online Supplement [file NIHMS1910530-supplement-Online_Supplement.docx]

**SUPPLEMENTARY TABLES**

**Supplementary Table A. Percentages of basket types by store type, country and region, Britain (2016, 2019)**

| **Area type** | **Stores (N)** | **Tobacco baskets** | | | | | | **Total baskets all four weeks** | |
| --- | --- | --- | --- | --- | --- | --- | --- | --- | --- |
|  |  | **Mean baskets per store per week** | | | **Percentage of total baskets** | | |  |  |
|  |  | **2016 (N)** | **2019 (N)** | **Change 2016-2019 (%)** | **2016 (%)** | **2019 (%)** | **Change 2016-2019 (%)** | **2016 (N)** | **2019 (N)** |
| **Total** | 1253 | 548.5 | 290.6 | -47.0 | 24.3 | 15.3 | -8.9 | 11,335,092 | 9,502,166 |
| **Store Type** |  |  |  |  |  |  |  |  |  |
| Independent | 390 | 476.0 | 246.1 | -48.3 | 24.2 | 15.3 | -8.9 | 3,069,611 | 2,513,427 |
| Symbol Group Franchise | 863 | 581.3 | 310.7 | -46.6 | 24.3 | 15.3 | -8.9 | 8,265,481 | 6,988,739 |
| **Country and Region** |  |  |  |  |  |  |  |  |  |
| England | 939 | 1463.9 | 775.5 | -47.0 | 24.3 | 15.3 | -8.9 | 22,670,184 | 19,004,332 |
| East Midlands | 108 | 542.4 | 280.5 | -48.3 | 25.3 | 16.1 | -9.2 | 926,858 | 753,265 |
| East of England | 125 | 473.0 | 247.5 | -47.7 | 21.5 | 13.4 | -8.0 | 1,102,173 | 920,491 |
| London | 24 | 523.8 | 262.0 | -50.0 | 22.7 | 14.6 | -8.1 | 221,332 | 172,031 |
| North East | 79 | 682.1 | 341.3 | -50.0 | 26.2 | 15.7 | -10.5 | 822,855 | 687,870 |
| North West | 100 | 669.7 | 338.8 | -49.4 | 26.1 | 16.0 | -10.1 | 1,024,933 | 845,734 |
| South East | 178 | 471.1 | 253.6 | -46.2 | 23.9 | 15.3 | -8.6 | 1,404,468 | 1,178,952 |
| South West | 119 | 415.5 | 227.6 | -45.2 | 19.0 | 12.1 | -6.8 | 1,042,425 | 892,655 |
| West Midlands | 118 | 536.1 | 280.0 | -47.8 | 24.8 | 15.7 | -9.1 | 1,020,820 | 841,135 |
| Yorkshire and The Humber | 88 | 593.4 | 299.3 | -49.6 | 24.3 | 14.6 | -9.7 | 858,432 | 720,214 |
| Scotland | 129 | 635.9 | 370.6 | -41.7 | 27.6 | 18.8 | -8.8 | 1,187,535 | 1,016,134 |
| Wales | 185 | 569.5 | 304.4 | -46.5 | 24.5 | 15.3 | -9.2 | 1,723,261 | 1,473,685 |

**Supplementary Table B. Linear regression of stores’ percentages of baskets containing tobacco by area deprivation and urban/rural status, Britain (2016, 2019)**

| **Area type** | **Percentage of store baskets containing tobacco** | | | | | | **Percentage change in store baskets containing tobacco** | | |
| --- | --- | --- | --- | --- | --- | --- | --- | --- | --- |
|  | **2016** | | | **2019** | | | **2016-2019** | | |
|  | **B** | **S.E.** | **p-value** | **B** | **S.E.** | **p-value** | **B** | **S.E.** | **p-value** |
| (Constant) | 15.3 | 0.8 | <0.000 | 11.6 | 0.7 | <0.000 | -3.7 | 0.5 | <0.000 |
| **Income Deprivation** |  |  |  |  |  |  |  |  |  |
| 1 Least deprived | - | - | - | - | - | - | - | - | - |
| 2 | 0.59 | 0.82 | 0.476 | -0.41 | 0.68 | 0.400 | -0.99 | 0.52 | 0.057 |
| 3 | 1.82 | 0.78 | 0.020 | 0.13 | 0.65 | 0.049 | -1.69 | 0.50 | 0.001 |
| 4 | 3.64 | 0.77 | <0.000 | 1.25 | 0.64 | 0.839 | -2.39 | 0.49 | <0.000 |
| 5 Most deprived | 3.71 | 0.76 | <0.000 | 0.53 | 0.63 | 0.550 | -3.18 | 0.48 | <0.000 |
| **Urban Rural Status** |  |  |  |  |  |  |  |  |  |
| Large Urban | 9.54 | 0.79 | <0.000 | 5.53 | 0.65 | <0.000 | -4.01 | 0.50 | <0.000 |
| Other Urban | 7.26 | 0.70 | <0.000 | 3.75 | 0.58 | <0.000 | -3.50 | 0.44 | <0.000 |
| Town Rural | 4.08 | 0.82 | <0.000 | 2.23 | 0.68 | 0.001 | -1.85 | 0.52 | <0.000 |
| Village Rural | - | - | - | - | - | - | - | - | - |
